# Bayesian Receiver Operating Characteristic Estimation of Multiple Tests for Diagnosis of Bovine Tuberculosis in Chadian Cattle

**DOI:** 10.1371/journal.pone.0008215

**Published:** 2009-12-09

**Authors:** Borna Müller, Penelope Vounatsou, Bongo Naré Richard Ngandolo, Colette Diguimbaye-Djaïbe, Irene Schiller, Beatrice Marg-Haufe, Bruno Oesch, Esther Schelling, Jakob Zinsstag

**Affiliations:** 1 Department of Public Health and Epidemiology, Swiss Tropical Institute, Basel, Switzerland; 2 Department of Biostatistics and Epidemiology, Swiss Tropical Institute, Basel, Switzerland; 3 Laboratoire de Recherches Vétérinaires et Zootechniques de Farcha, N'Djaména, Chad; 4 Prionics AG, Schlieren-Zurich, Switzerland; Charité-Universitätsmedizin Berlin, Germany

## Abstract

**Background:**

Bovine tuberculosis (BTB) today primarily affects developing countries. In Africa, the disease is present essentially on the whole continent; however, little accurate information on its distribution and prevalence is available. Also, attempts to evaluate diagnostic tests for BTB in naturally infected cattle are scarce and mostly complicated by the absence of knowledge of the true disease status of the tested animals. However, diagnostic test evaluation in a given setting is a prerequisite for the implementation of local surveillance schemes and control measures.

**Methodology/Principal Findings:**

We subjected a slaughterhouse population of 954 Chadian cattle to single intra-dermal comparative cervical tuberculin (SICCT) testing and two recently developed fluorescence polarization assays (FPA). Using a Bayesian modeling approach we computed the receiver operating characteristic (ROC) curve of each diagnostic test, the true disease prevalence in the sampled population and the disease status of all sampled animals in the absence of knowledge of the true disease status of the sampled animals. In our Chadian setting, SICCT performed better if the cut-off for positive test interpretation was lowered from >4 mm (OIE standard cut-off) to >2 mm. Using this cut-off, SICCT showed a sensitivity and specificity of 66% and 89%, respectively. Both FPA tests showed sensitivities below 50% but specificities above 90%. The true disease prevalence was estimated at 8%. Altogether, 11% of the sampled animals showed gross visible tuberculous lesions. However, modeling of the BTB disease status of the sampled animals indicated that 72% of the suspected tuberculosis lesions detected during standard meat inspections were due to other pathogens than *Mycobacterium bovis*.

**Conclusions/Significance:**

Our results have important implications for BTB diagnosis in a high incidence sub-Saharan African setting and demonstrate the practicability of our Bayesian approach for diagnostic test evaluation.

## Introduction

*Mycobacterium bovis* is the causative agent of bovine tuberculosis (BTB) and belongs to the *Mycobacterium tuberculosis* complex (MTBC) of bacteria [1]. BTB is a major problem in developing countries, which bear the largest part of the world-wide disease burden and where millions of people are affected by neglected zoonotic diseases such as BTB [2]–[5]. The disease causes economic loss by its effects on animal health and productivity and by international trade restrictions [6]. It can also affect health of wildlife [7] and infected wildlife populations serve as reservoirs and hamper disease eradication programs in several countries [8]. Moreover, *M. bovis* infections are of public health concern due to the pathogen's zoonotic potential [2], [3].

BTB control and surveillance is scarce in sub-Saharan Africa and mostly limited to abattoir meat inspections. However, the performance of meat inspection is rather poor and depends on the disease stage in which infected animals reside, the accuracy of the carcass examination and the presence of other lesion causing pathogens [9]–[13]. Recent studies have detected a high proportion of non-tuberculous mycobacteria (NTM) in lesions from Chadian, Ugandan, Ethiopian and Sudanese cattle, suggesting that a considerable amount of lesions detected during abattoir meat inspection of African cattle might be due to other bacteria than *M. bovis* [14]–[17].

Current ante mortem diagnosis of BTB mainly relies on the single intra-dermal comparative cervical tuberculin (SICCT) test, which, although imperfect, could not yet be replaced by any other more accurate diagnostic method [13]. SICCT is based on the cell mediated immune (CMI) response against tuberculosis infection. TB in cattle is characterized by an early Th1 type CMI response, whilst humoral immune responses develop as disease progresses. At late disease stages, the CMI response can decrease and SICCT anergic animals can show false negative test results [13], [18], [19]. Moreover, SICCT performance is influenced by animal exposure to NTM strains as their antigens can cross-react with tuberculin [13]. Serological tests detecting humoral immune responses may be more useful to detect late stage diseased animals. Fluorescence polarization assays (FPA) constitute a technique for antibody detection with a shown potential for diagnostic purposes [20]. An assay for the detection of *M. bovis* antibodies has been described recently [21]–[25].

Attempts to evaluate diagnostic tests for BTB in naturally infected cattle in Africa are scarce but a prerequisite for the implementation of surveillance schemes and control measures. Gobena et al. have used detailed post mortem examination to define the BTB disease status of Ethiopian cattle for the evaluation of SICCT in this setting [26]. However, due to the generally low sensitivity and specificity of post mortem meat inspection, its use as a gold standard test is not ideal [12]. We have recently assessed three different tests for the diagnosis of BTB (SICCT and two newly developed FPA methods) in Chadian cattle. Our previous evaluation was also based on a gold standard approach using PCR confirmed MTBC infected and lesion negative animals as the positive and negative population, respectively [25]. Drawbacks of this study were the small number of positive animals and the unknown true disease status of the lesion negative cattle.

Choi et al. [27] developed a Bayesian model for the receiver operating characteristic (ROC) estimation of two diagnostic tests in the absence of a gold standard test. In the present study, we have further extended this model and applied it to evaluate the performance of the diagnostic tests previously assessed by the gold standard approach [25]. Our Bayesian model integrated information from three different diagnostic methods and was independent of a gold standard test; moreover, it allowed us to estimate the true BTB prevalence in the sampled population and the true disease status of each tested animal. Using this information, we could in addition calculate the diagnostic errors of four post-mortem tests (meat inspection, microscopic examination of BTB-like lesions, microscopic examination of derived bacterial cultures and PCR on microscopy positive cultures).

## Results

### Test results

A total number of 954 sequentially selected slaughter animals from Southern Chad were subjected to multiple tests for the diagnosis of BTB. Three ante-mortem tests with continuous numerical outcome values (continuous outcome) were used, namely, SICCT and two recently developed FPA tests termed SENTRY 100 and GENios Pro [25]. Also, four post-mortem tests giving either a positive or negative test result (binary outcome) were applied. These tests were the post-mortem meat inspection, direct microscopy, culture and microscopy and PCR (see materials and methods for details on the applied tests).

Before slaughter, blood samples were collected and animals underwent SICCT testing. Altogether, 8% (CI: 6%–10%) of the animals tested, reacted positively to SICCT when the official OIE cut-off (>4 mm; [28]) was used (Table 1). Serum extracted from the blood samples was subjected to the FPA tests SENTRY 100 and GENios Pro, for which we have determined most appropriate cut-off values within this study (results shown below; Table 1). After slaughter, cattle carcasses underwent meat inspection; lesions suggestive of tuberculosis were isolated from 108 animals (lesion prevalence: 11%; CI: 9%–14%; Table 1). In lesions of 51 animals (47% of animals with lesions; CI: 38%–57%), acid-fast bacilli (AFB) were observed by direct microscopy (Table 1). Culture of lesions and subsequent microscopic examination detected AFB in samples from 50 animals (49% of the animals tested; CI: 39%–59%; Table 1). The microscopy results obtained before and after culture agreed by 86%. In AFB containing cultures of 20 animals MTBC strains could be detected by real-time PCR (Table 1). In cultures of 13 animals, NTM strains were detected; three of which showed a mixed infection with MTBC strains.

**Table 1:** Tests applied for the diagnosis of BTB in Chadian cattle.

Test	No. of animals tested	Outcome	Ante-/post mortem	No. of animals tested pos.	% pos.
SICCT (OIE cut-off>4 mm)*	930	continuous	ante-mortem	72	7.7%
SICCT (cut-off>2 mm)*	930	continuous	ante-mortem	144	15.5%
SENTRY 100 (cut-off≥15 ΔmP)*	953	continuous	ante-mortem	62	6.5%
GENios Pro (cut-off≥38 ΔmP)*	954	continuous	ante-mortem	119	12.5%
Meat inspection	954	binary	post-mortem	108	11.3%
Direct microscopy	108	binary	post-mortem	51	47.2%
Culture and microscopy	102	binary	post-mortem	50	49.0%
PCR	50	binary	post-mortem	20	40.0%

% pos.: Number of animals tested positive divided by the total number of animals subjected to the respective test.

* SICCT, SENTRY 100 and GENios Pro results without missing data were available for 929 animals.

### Model selection

Based on the same data, we have previously reported the evaluation of SICCT, SENTRY 100 and GENios Pro using a subset of animals with either PCR confirmed MTBC infections or no visible lesions [25]. Drawbacks of this approach were the small number of positive animals and the uncertainty about the true disease status of lesion negative animals [25]. The latter is due to the fact that no gross lesions may be observed at early stages of BTB. Here, we describe a Bayesian method for the estimation of the true disease prevalence in the sampled population and the means and variance-covariances of SICCT, SENTRY 100 and GENios Pro test outcomes for the diseased and non-diseased animals. In an initial model we have included data from the post-mortem tests with binary outcomes and attempted to directly estimate their sensitivities and specificities. Prior assumptions and model estimates are indicated in Table S1 (models 1A and 1B).

Model estimates for tests with binary outcome were highly sensitive to the priors. We therefore decided to consider solely tests with continuous outcome for Bayesian modeling (model 2A and 2B; see Table S1). Parameter estimations for these tests did not appear to be sensitive to the prior assumptions and were only marginally different in models 1A, 1B, 2A and 2B (see Table S1).

### Diagnostic test performances

**Figure 1:**
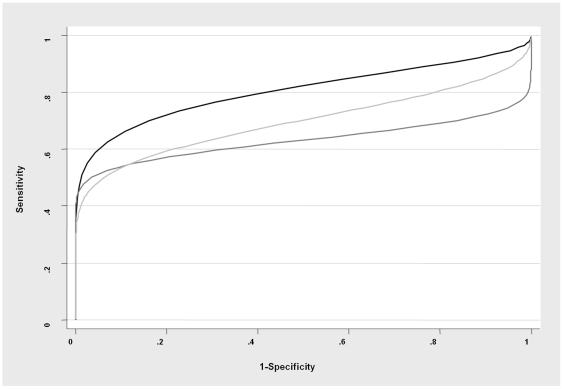
Calculated ROC curves for SICCT (black), SENTRY 100 (dark gray) and GENios Pro (light gray).

Based on the estimates for the means and variance-covariances of SICCT, SENTRY 100 and GENios Pro test results for the diseased and non-diseased animals in model 2A (see Table S1), ROC curves were calculated for each test (Fig. 1) and the most appropriate cut-off for positive test interpretation was defined as the point from the ROC curve with the largest distance from the diagonal line (sensitivity = 1−specificity). For SICCT, a cut-off greater than 2 mm (>2 mm) appeared to be most appropriate for our setting. For SENTRY 100 and GENios Pro the best cut-off values were determined at 15 ΔmP (≥15 ΔmP) and 38 ΔmP (≥38 ΔmP), respectively. Using these values, the sensitivities and specificities of the tests were calculated (Table 2). The prevalence of *M. bovis* infection in the sampled population was estimated at 8% (CI: 6%–11%).

**Table 2:** Parameter estimates for different diagnostic tests based on results from model 2A (see Table S1).

Test	AUC	95% CI	S	95% CI	C	95% CI
SICCT (OIE cut-off>4 mm)	0.80	0.73–0.87	51.1%	42.1–60.1%	98.6%	97.9–99.2%
SICCT (cut-off>2 mm)	0.80	0.73–0.87	66.3%	57.5–74.6%	89.2%	86.6–91.5%
SENTRY 100 (cut-off≥15 ΔmP)	0.57	0.51–0.65	45.5%	39.3–52.9%	96.4%	95.4–97.4%
GENios Pro (cut-off≥38 ΔmP)	0.64	0.57–0.72	47.2%	39.9–54.7%	92.4%	90.7–93.9%
Meat inspection*	-	-	36.1%	26.6–46.9%	90.8%	88.6–92.5%
Direct microscopy*	-	-	90.0%	74.4–96.5%	66.7%	55.2–76.5%
Culture and microscopy*	-	-	93.3%	78.6–98.2%	69.4%	58.0–78.8%
PCR*	-	-	71.4%	52.9–84.7%	100.0%	85.1–100%
True prevalence	8.4%	6.1–11.0%				

AUC: area under the ROC curve; CI: confidence interval; S: sensitivity; C: specificity.

* Estimates are based on modeled latent disease state of the animals and refer to the sample; 95% CI are Wilson confidence intervals.

In addition to the parameters described above, Bayesian modeling allowed us to compute the latent disease status of the sampled animals. We have used this information from model 2A (see Table S1) to calculate the sensitivities and specificities of the post-mortem tests with binary outcome to detect modeled *M. bovis* infected animals (Table 2). It must be noted, that these estimates refer to the diagnostic performance for our sample, whereas the Bayesian model estimates consider the fact that our sample was a sub-population of the general slaughterhouse population.

Surprisingly, 72% of the animals with gross visible tuberculous lesions detected during standard meat inspection showed a negative result for modeled *M. bovis* infection. Thus, our analysis suggested that 72% of the animals exhibiting tuberculosis-like lesions were infected with other pathogens than *M. bovis*.

### Risk factors

We performed logistic regression to identify risk factors for modeled *M. bovis* infection. Univariate logistic regression with modeled *M. bovis* infection as outcome variable and age, sex, animal breed and body condition as explanatory variables identified age and a very bad body condition as risk factors for modeled *M. bovis* infection (Table 3). However, in the multiple model, only age turned out to be significantly associated with modeled *M. bovis* infection (Table 3). Interestingly, only the presence of organ lesions in general and in particular the presence of lung and liver lesions was significantly associated with modeled *M. bovis* infection (Table 4). The presence of lymph node lesions was not associated with modeled *M. bovis* infection (Table 4).

**Table 3:** Logistic regression with modeled *M. bovis* infection as outcome variable and age, sex, breed and body condition as explanatory variables.

Explanatory variable		Univariate model			Multiple model*		
Category	Subcategory	OR	95% CI	p	OR	95% CI	p
Age		1.15	1.05–1.26	<0.01	1.14	1.02–1.29	<0.05
Sex		1.59	0.96–2.64	0.07	1.11	0.61–2.01	0.74
Breed		1.28	0.79–2.06	0.31	1.54	0.94–2.54	0.09
Body condition							
	good	1.00	-	-	1.00	-	-
	bad	1.07	0.66–1.73	0.79	0.96	0.58–1.58	0.86
	very bad	2.81	1.33–5.95	<0.01	1.96	0.88–4.38	0.10

OR: odds ration; CI: confidence interval; p: p-value.

* The multiple model was adjusted for age, sex, breed and body condition.

**Table 4:** Lesion distribution and association between lesion location and modeled *M. bovis* infection.

	N	%	RR	Fisher
**Animals with lesions**	**108**	**100%**	**N/A**	**N/A**
***Lymph node lesions***	***98***	***91%***	***0.66***	***0.46***
Pre-scapular lymph nodes	64	59%	1.19	0.67
Mammary lymph nodes	37	34%	1.11	0.82
Head associated	8	7%	0.43	0.44
Popliteal lymph nodes	1	1%	0.00	1.00
***Organ lesions***	***22***	***20%***	***2.99***	***<0.01***
Lung	17	16%	3.10	<0.01
Liver	8	7%	2.50	<0.04
Others	3	3%	2.50	0.19

N: Number of animals with lesions at the specified location. %: Percentage of animals with lesions at the specified location. RR: Risk ratio for modeled *M. bovis* infection. Fisher: Fisher's exact test p-value.

## Discussion

### Practicability and significance of Bayesian ROC estimation

The performance of diagnostic tests is often setting dependent [29]. Thus, evaluations of diagnostic tests for a given region are a prerequisite for the implementation of local disease surveillance schemes and control measures [29]. However, to date, only few studies have assessed the performance of tests for the diagnosis of BTB in high incidence countries in Africa. Furthermore, test evaluation is hampered by the absence of a gold standard method for the identification of the animal's true disease status. Here, we applied a Bayesian approach for the evaluation of multiple tests for the diagnosis of BTB in a naturally infected slaughterhouse population of cattle in Southern Chad. Our approach did not require knowledge of the true disease status of the tested animals. Moreover, it allowed the estimation of the true disease prevalence in the sampled population, the calculation of the BTB disease status of all sampled animals and the evaluation of four post-mortem tests for the diagnosis of BTB.

We have previously reported the evaluation of SICCT, SENTRY 100 and GENios Pro using a subset of the same data [25]. In a gold standard approach, PCR confirmed MTBC infected animals were defined as the positive population and lesion negative animals as the negative population and used for the construction of ROC curves for each test. Drawbacks of this approach were the relatively small amount of confirmed infections and the unknown true disease status of lesion negative animals. Table 5 compares the results from the present and our previously published study [25]. The accordance of our results using the two different approaches further supports the accuracy of our estimates and the practicability of our Bayesian method. Noteworthy, Bayesian modeling gave rise to parameter estimates with in many cases considerably smaller confidence intervals compared to the gold standard approach (Table 5).

**Table 5:** Comparison of parameter estimates derived from the herein described Bayesian model and from a previously applied gold standard approach [25].

Cut-off	SICCT		SENTRY 100	GENios Pro
	>4mm	>2mm	≥15	≥38
*Bayesian method:*				
Sensitivity	51.1% (42.1–60.1%)	66.3% (57.5–74.6%)	45.5% (39.3–52.9%)	47.2% (39.9–54.7%)
Specificity	98.6% (97.9–99.2%)	89.2% (86.6–91.5%)	96.4% (95.4–97.4%)	92.4% (90.7–93.9%)
AUC	0.80 (0.73–0.87)	0.80 (0.73–0.87)	0.57 (0.51–0.65)	0.64 (0.57–0.72)
*Gold standard approach:*				
Sensitivity	20.0% (5.7–43.7%)*	65.0% (43.3–81.9%)	30.0% (14.5–51.9%)	50.0% (29.9–70.1%)
Specificity	93.1% (91.1–94.6%)	86.7% (84.2–88.9%)	94.4% (92.7–95.8%)	88.4% (86.1–90.4%)
AUC	0.80 (0.71–0.88)	0.80 (0.71–0.88)	0.70 (0.58–0.82)	0.67 (0.52–0.82)

The previously conducted diagnostic test evaluation considered animals with PCR confirmed infections and animals not showing lesions during post mortem meat inspection as disease positive and negative animals, respectively.

* 95% binomial exact confidence intervals are indicated because (estimated value)×(sample size)≤5; for all other parameter estimates in the gold standard approach, Wilson confidence intervals are shown.

### SICCT

Our results indicated that the most appropriate cut-off for positive SICCT test interpretation was significantly lower then the OIE suggested standard cut-off (>2 mm versus >4 mm). However, our criteria for cut-off selection attributed equal weights to sensitivity and specificity and did not consider the disease prevalence and the cost of misclassifications. As an alternative approach for cut-off selection, the misclassification-cost term (MCT) can be calculated for each point of the ROC curve. The point with the lowest MCT value would then be most appropriate for positive test interpretation [30]. This method requires to quantify the cost of false negative (C_FN_) and false positive (C_FP_) diagnosis, which we were not able to accurately do. However, the cost of a false negative diagnosis is likely to exceed the cost of a false positive result by several folds as disease transmission amplifies the total economical losses due to BTB. We found that, assuming a disease prevalence of 8.4% (10.0%), a cut-off >2 mm would be ideal if C_FN_/C_FP_ lies between 8 and 16 (7 and 13). This suggests that our chosen cut-off values may be acceptable for a broad range of reasonable C_FN_/C_FP_ ratios.

A cut-off >2 mm was also found to be most appropriate for positive SICCT test interpretation in a recent study in Ethiopia [26] and in SICCT reactor prevalence studies in Uganda and Tanzania, lower cut-offs than the OIE standard cut-off have been used, however without detailed justification [31], [32]. Accordingly, our results are likely to apply for many other countries in sub-Saharan Africa with similar environmental and economic conditions.

SICCT showed a relatively low sensitivity irrespective of whether our suggested or the OIE cut-off was used (Table 2). Comparable results were obtained in previous studies in Ireland and Madagascar [13], [33]. This relatively weak performance may be explained by several factors. A high proportion of pre-allergic animals at an early stage of BTB infection or a high amount of SICCT anergic animals at a very late disease stage could have accounted for this observation [13]. Antigens of co-infecting NTM strains, cross reacting with PPD-A could also cause false negative test results as well as nutritional stress or concurrent infections with pathogens leading to immuno-depression [13]. For SICCT anergy due to generalized BTB, one would expect the presence of gross visible lesions. Amongst all animals with a modeled *M. bovis* infection and visible lesions (N = 30), 9 or 19 (30% or 63%) did not show a positive reaction to SICCT depending on whether a cut-off >2 mm or >4 mm was applied, respectively. This indicates a considerable proportion of SICCT anergic animals (9 or 19 of altogether 83 animals with modeled *M. bovis* infection). Unfortunately, our sample size was too small to conclusively assess the ability of the FPA tests to detect such animals.

### Cause of lesions

Our data suggests that a surprisingly high proportion of lesions detected during standard meat inspection at the Sarh abattoir in Southern Chad was caused by other bacteria than *M. bovis*. For 72% of the animals in which lesions have been detected, no *M. bovis* infection was modeled. This finding was in line with the relatively low amount of MTBC strains detected in animals with lesions (20 of altogether 108 animals with lesions; Table 1). Interestingly, modeled *M. bovis* infection was only significantly associated with organ lesions in general and the presence of lung and liver lesions in particular (Table 4). The presence of lymph node lesions was not associated with modeled *M. bovis* infection (Table 4). Altogether, this suggests that a significant amount of gross visible lesions detected during standard meat inspection at the Sarh abattoir has been caused by other pathogens than *M. bovis* and that especially a large proportion of the detected lymph node lesions may have been caused by these pathogens.

NTM infections without concomitant *M. bovis* infections have been isolated from 10 out of 50 animals tested by PCR. This could indicate that some of the lesions may have been associated with NTMs. This is also supported by the comparatively low specificity of Ziehl-Neelsen staining and microscopic examination of extracted lesions or bacterial cultures in our setting compared to previous studies (Table 2) [34]–[38]. Nevertheless, the low amount of cultures in which AFB have been detected (50 of 108 animals with lesions) suggests that in addition, other pathogens may have been responsible for the detected lesions.

Altogether, our data indicates that the amount of gross visible granulomatous lesions caused by other pathogens than *M. bovis* may be greatly underestimated in this setting. Low recovery of *M. bovis* from cultures of granulomatous lesions have been reported in several studies on BTB in sub-Saharan Africa [16], [17], [39], [40]. It is conceivable that in many of these cases, lesions may have been caused by other pathogens and that these bacteria may have remained undetected e.g. due to the decontamination procedure or different culture growth requirements.

However, it has to be noted that the proportion of lesions due to other pathogens than *M. bovis* is dependent on the accuracy of the meat inspection. Inaccurate meat inspection e.g. biased toward superficial lymph nodes could have distorted the relative proportion of lesions found in different organs. In particular, it is surprising that no lesions were detected in the bronchial or mediastinal lymph nodes, as these are usually the most often affected tissues in bovine tuberculosis [9], [10], [41], [42]. Also, the sensitivity of meat inspection to detect *M. bovis* infected cattle was lower in our setting compared to the results of previous studies [9]–[12]. Therefore, the proportion of lesions caused by other pathogens than *M. bovis* may have to be interpreted with caution.

### Risk factors

In a previous study on BTB in Chadian cattle we have reported that the prevalence of BTB was significantly higher in Mbororo zebus than in Arab zebus [40]. Our results from the logistic regression analysis could not show any evidence that *M. bovis* infection was significantly associated with breed (Table 3). Nevertheless, the presence of lesions was still significantly associated with Mbororo zebus (N = 944, χ^2^ = 5.23, p = 0.02). This observation could suggest that Mbororo breeds in fact, are not more likely to be infected with *M. bovis* but more often develop advanced stages of the disease. Host genetic factors as well as environmental factors or animal husbandry could account for this observation.

### Conclusions

In summary, the present study shows the practicability of a Bayesian method for the evaluation of multiple tests for the diagnosis of BTB in naturally infected cattle and in absence of knowledge of the true disease status of the animals. Our model allowed us to compute the disease status of each sampled animal and the modeling results supported our previous observation that the cut-off for positive SICCT interpretation should be lowered to >2 mm in many countries of sub-Saharan Africa. Moreover, we provide evidence that an unexpectedly high proportion of BTB suspect lesions detected during slaughterhouse meat inspection was due to other pathogens than *M. bovis*.

## Materials and Methods

### Animals

The animal population subjected to this study has previously been described, in detail [25]. A total of 954 slaughter animals were sampled during three intervals of approximately one month between July and November 2005 at abattoirs in Southern Chad. We can assume that the tested animals constitute a representative sample of slaughter cattle from a large number of different herds and a big area in Southern Chad [25]. Presumably, none of the animals has ever undergone tuberculin skin testing. Four types of phenotypic zebu breeds were encountered: Arab (N = 658), Mbororo (N = 286), Bogolodjé (N = 7) and cross breeds (N = 3).

### Physical examination of animals

All 954 animals were physically examined before slaughter. Body condition was assessed by assigning one of the following three scores: 1 – good body condition, 2 – bad body condition, 3 – very bad body condition [25].

### Test procedures

#### *SICCT*

Valid SICCT testing results were available for 930 animals. SICCT testing and reading was carried out as explained previously and according to standard protocols [25], [28].

#### *Fluorescence polarization assays*

Valid SENTRY 100 and GENios Pro FPA results were available for 953 and 954 animals, respectively. The methods have been previously described in detail [21], [25].

#### *Meat inspection*

After slaughter, all 954 animals underwent meat inspection, which included organ and lymph node palpation, visual inspection and incision of organs and lymph nodes according to standard procedures [43]. However, we were not able to fully exclude potential irregularities during the carcass examinations. Meat inspection was done by local meat inspectors at the abattoirs in Southern Chad. Gross visible lesions were detected in altogether 108 of the 954 sampled animals. Lesion containing tissue specimens from all visibly affected organs and lymph nodes were collected and transported on ice to the Chadian National Veterinary and Animal Husbandry Laboratory (Laboratoire de Recherches Vétérinaries et Zootéchniques de Farcha) in N'Djaména and stored at −20°C.

#### *Direct microscopy*

Specimens from all 108 animals with lesions were subjected to direct microscopy and processed as previously described [40]. After homogenisation, specimens were colorized by Ziehl-Neelsen staining and examined under the light-microscope for the presence of Acid-Fast Bacilli (AFB). The samples were decontaminated with N-acetyl-L-cysteine sodium hydroxide (0.5% NALC 2% NaOH) and again examined for the presence of AFB under the microscope. If either of the two microscopic examinations revealed presence of AFB the result was considered to be positive.

#### *Culture and microscopy*

Specimens of lesions from altogether 102 animals were subjected to culture and microscopy. Decontaminated samples were inoculated into two Middlebrook 7H9 medium flasks containing OADC and PANTA and either glycerol (0.75%) or pyruvate (0.6%) [25]. Samples were put into culture until growth was detected or for a minimum of 8 weeks. Presence of AFB in cultures was examined by Ziehl-Neelsen staining and microscopy [40]. Bacterial growth was detected in cultures of 102 animals; cultures of 50 animals showed presence of AFB by Ziehl-Neelsen staining.

#### *Real-time PCR*

AFB containing cultures from 50 animals were subjected to molecular characterization. Heat inactivation of the cultures was carried out as previously explained [25]. Thermolysates were shipped to the Swiss Reference Centre for Mycobacteria, DNA was extracted by means of the InstaGene™ Matrix (Bio-Rad) and identification of MTBC and NTM strains was carried out by means of Light Cycler® PCR as previously described by Lachnik et al. [44].

### Statistical analyses

A Bayesian model was developed to estimate the true *M. bovis* infection status of all sampled animals. The model combined the results of the continuous as well as the binary diagnostic tests for BTB (SICCT, SENTRY 100, GENios Pro, meat inspection, direct microscopy, culture and microscopy and PCR), applied to the same animal population without considering a gold standard. It also allowed the estimation of the true disease prevalence in the sampled population as well as the sensitivities and specificities of the diagnostic tests. A mathematical description of the model and the WinBUGS code are provided in Text S1 and Text S2, respectively.

Risk factors for the modeled *M. bovis* infection status were identified by univariate and multiple logistic regression analysis in Stata (Stata/IC v10.0). Association between lesion localisation and modeled *M. bovis* infection was assessed by the Fisher's exact test in Stata.

#### *Bayesian modeling of disease status*

We assumed that the distribution of the test values of SICCT, SENTRY 100 and GENios Pro was trivarite normal with means and variance-covariances separately estimated for the diseased and the non-diseased animals. The normality assumption was verified via the shape of the histogram of the test values. In an initial model we have included the data from the multiple post mortem tests for the detection of *M. bovis* infected animals (meat inspection, direct microscopy, culture and microscopy and PCR) and tried to directly model their sensitivities and specificities (models 1A and 1B, see Table S1). Because the parameter estimations for the binary post-mortem tests were highly sensitive to the prior assumptions [9]–[12], [34]–[38], [45], [46], we eventually excluded the respective data from the Bayesian model formulation (models 2A and 2B, Table S1). In order to estimate the performance of the binary tests, we used the modeled latent *M. bovis* infection status of each animal to calculate sensitivities and specificities of the respective tests. Model fit was done in the statistical package WinBUGS (Imperial College and Medical Research Council, UK). The mathematical description of the model and the WinBUGS code are shown in Text S1 and Text S2. Real-time PCR was assumed to be 100% specific and animals with a positive PCR test outcome were therefore defined as MTBC infected in all Bayesian models.

#### *ROC curve and cut-off selection*

From the estimates of the means and variance-covariances of the multivariate normally distributed continuous test values of SICCT, SENTRY 100 and GENios Pro for the diseased and non-diseased animals, a ROC curve was calculated in Stata. Pairs of 1-specificity and sensitivity were calculated and plotted for all possible cut-off points according to the following formula:
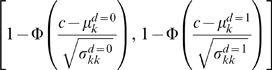
Φ is the cumulative distribution of a standard normal variable; c is the cut-off value; 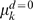 and 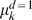 are the means of the multivariate normal distribution of the test values for the non-diseased (d = 0) and the diseased (d = 1) animal population subjected to test *k* (*k* = 1 for SICCT, *k* = 2 for SENTRY 100, *k* = 3 for GENios Pro), respectively; 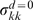 and 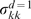 are the variance of the non-diseased and the diseased animal population subjected to test *k*, respectively.

We considered the point of the ROC plot with the greatest distance from the diagonal line (sensitivity = 1−specificity) as the best cut-off; this corresponds to the point with the largest Youden index (J = sensitivity+specificity−1) [30], [47]. In cases where several points showed the same distance, the point with the highest sensitivity was chosen. For cut-off selection using the misclassification-cost term (MCT), the point with the smallest MCT value [MCT = (C_FN_/C_FP_) P (1−Se)+(1−P) (1−Sp)] was chosen, with C_FN_ and C_FP_ being the cost of false negative and false positive diagnosis, respectively and P being the disease prevalence in the target population [30]. We were unable to accurately estimate C_FN_/C_FP_ but the cost of false-negative diagnosis is likely to exceed the cost of false positive diagnosis. Therefore, MCT values for each possible cut-off point and different ratios of C_FN_/C_FP_ were calculated and compared, assuming a disease prevalence of 8.4%, as estimated by our model. In addition, MCT values for different C_FN_/C_FP_ ratios were calculated for a 10.0% disease prevalence.

## Supporting Information

Table S1 Priors and model estimates for different parameters. μ^d^: Mean diagnostic value for non-diseased (d = 0) and diseased (d = 1) animals, respectively. τ^d^: precision of the diagnostic values for non-diseased (d = 0) and diseased (d = 1) animals, respectively. m^S^/σ^S^, m^C^/σ^C^, m_π_/σ_π_ : Mean and standard deviation of the test sensitivity, specificity and true disease prevalence, respectively. The normal distribution is parametrized in terms of mean and variance. The Gamma distribution is parametrized in a non-conventional way in terms of mean and variance instead of the shape and scale parameters. Model-based estimates correspond to the posterior mean and standard deviation in brackets.
(0.04 MB XLS)

Text S1 Mathematical model description
(0.12 MB DOC)

Text S2 WinBUGS code
(0.03 MB DOC)
