# Supplementary material for: Bayesian Receiver Operating Characteristic Estimation of Multiple Tests for Diagnosis of Bovine Tuberculosis in Chadian Cattle
Source: PLoS One. 2009 Dec 9;4(12):e8215. doi: 10.1371/journal.pone.0008215 (PMC2785429; doi:10.1371/journal.pone.0008215)
Supplement: Text S1 — Mathematical model description (0.12 MB DOC) [file pone.0008215.s002.doc]

**Text S1 – Mathematical model description**

Let $Y_{ki}^{d}$ denote the diagnostic test values of the *i*th animal, *i*= 1,…,*m* with disease status *d* (*d* = 0 for non-diseased; *d* = 1 for diseased) for test *k* (*k* = 1 for SICCT; *k* = 2 for SENTRY 100; *k* = 3 for GENios Pro). We assume that the test scores of the three tests are multivariate normally distributed, that is $Y_{i}^{d}=(Y_{1i}^{d},Y_{2i}^{d},Y_{3i}^{d})^{T}\sim MVN(\mathbf{\mu}_{i}^{d},\Sigma^{d})$ where $\mathbf{\mu}_{i}^{d}=(\mu_{i1}^{d},\mu_{i2}^{d},\mu_{i3}^{d})^{T}$ and $\Sigma_{kl}^{d}=\mathrm{cov}(Y_{ik}^{d},Y_{il}^{d})=\sigma_{kl}^{d}$ with *k, l* = 1,2,3. We write the likelihood as a product of the following factors:

$L(Y_{i};)\infty\prod_{d=0,1} \prod_{i=1}^{n_{d}} f\left(Y_{i3}^{d}|Y_{i2}^{d},Y_{i1}^{d};\mu_{i}^{d},\left\{\sigma_{kl}^{d}\right\}\right)f\left(Y_{i2}^{d}|Y_{i1}^{d};\mu_{i}^{d},\left\{\sigma_{kl}^{d}\right\}\right)f\left(Y_{i1}^{d};\mu_{i}^{d},\left\{\sigma_{kl}^{d}\right\}\right)$,

which have normal distributions with

$Ef\left(Y_{i1}^{d};\mu_{i}^{d},\left\{\sigma_{kl}^{d}\right\}\right)=\mu_{i1}^{d}$ and $\mathrm{var}f\left(Y_{i1}^{d};\mu_{i}^{d},\left\{\sigma_{kl}^{d}\right\}\right)=\sigma_{11}^{d}$,

$Ef\left(Y_{i2}^{d}|Y_{i1}^{d};\mu_{i}^{d},\left\{\sigma_{kl}^{d}\right\}\right)=\mu_{i2}^{d}+\rho_{12}^{d}\sqrt{\frac{\sigma_{22}^{d}}{\sigma_{11}^{d}}}\left(Y_{i1}^{d}-\mu_{i1}^{d}\right)$ and $\mathrm{var}f\left(Y_{i2}^{d}|Y_{i1}^{d};\mu_{i}^{d},\left\{\sigma_{kl}^{d}\right\}\right)=\sigma_{22}^{d}\left(1-\left(\rho_{12}^{d}\right)^{2}\right)$ and

$\begin{matrix} Ef\left(Y_{i3}^{d}|Y_{i2}^{d},Y_{i1}^{d};\mu_{i}^{d},\left\{\sigma_{kl}^{d}\right\}\right)=\mu_{i3}^{d}+\left(\sigma_{11}^{d}\sigma_{22}^{d}\left(1-\left(\rho_{12}^{d}\right)^{2}\right)\right)^{-1}\times\\ \times\left\{\left(\rho_{32}^{d}+\rho_{12}^{d}\rho_{31}^{d}\right)\sigma_{11}^{d}\sqrt{\sigma_{22}^{d}\sigma_{33}^{d}}\left(Y_{i2}^{d}-\mu_{i2}^{d}\right)+\left(\rho_{31}^{d}+\rho_{12}^{d}\rho_{32}^{d}\right)\sigma_{22}^{d}\sqrt{\sigma_{11}^{d}\sigma_{33}^{d}}\left(Y_{i1}^{d}-\mu_{i1}^{d}\right)\right\} \end{matrix}$and

$\mathrm{var}f\left(Y_{i3}^{d}|Y_{i2}^{d},Y_{i1}^{d};\mu_{i}^{d},\left\{\sigma_{kl}^{d}\right\}\right)=\sigma_{33}^{d}\left(1-\left(\left(\rho_{32}^{d}\right)^{2}+\left(\rho_{31}^{d}\right)^{2}\right)\left(1-\left(\rho_{12}^{d}\right)^{2}\right)^{-1}\right)$.

$\rho_{kl}^{d}\text{ }$is a correlation parameter between tests *k* and *l* and it is defined in terms of the variances as follows: $\rho_{kl}^{d}\text{ }=\text{ }\frac{\sigma_{kl}^{d}}{\sqrt{\sigma_{kk}^{d}}\sqrt{\sigma_{ll}^{d}}}$.

The AUCs for each diagnostic test *k* (*k* = 1, 2, 3) can be calculated as

$AUC_{k}\text{ }=\text{ }\Phi\left(-\frac{\mu_{k}^{d=0}-\mu_{k}^{d=1}}{\sqrt{\sigma_{kk}^{d=0}+\sigma_{kk}^{d=1}}}\right)$ with $\Phi$ being the cumulative distribution function of a standard normal variable. For identifiability we assume that $\mu_{k}^{d=0}<\mu_{k}^{d=1}$, which holds for our diagnostic tests.

In an initial model, in addition to both FPA methods and SICCT, we have taken into account the results of a number of binary tests (meat inspection, direct microscopy, culture and microscopy, PCR), assuming that they had imperfect sensitivity and specificity, with the exception of PCR, which has been considered to be 100% specific. Let *π* be the unknown true disease prevalence in the sampled population and $p_{j}$ the observed prevalence estimated from the *j*th test (*j*= 1 for meat inspection; *j*= 2 direct microscopy; *j*= 3 for culture and microscopy; *j*= 4 for PCR). Moreover, let *T_i_* be the latent variable that indicates the true disease status of the *i*th animals (1 for diseased and 0 for non-diseased animals) and let *Z_ij_* be the observed disease status from the *j*th test. We assume that $Z_{ij}\text{ }\sim\text{ }Bernoulli(p_{j})$, $T_{i}\text{ }\sim\text{ }Bernoulli(\pi)$ and define *TT_i_* where $TT_{i}\text{ }=\text{ }\left\{\begin{matrix} T_{i}\text{ if PCR result is negative} \\ 1\text{ if PCR result is positive} \end{matrix}\right\}$ as we consider positive values of PCR to be the gold standard and $p_{j}=S_{j}\pi+(1-C_{j})(1-\pi)$ where $S_{j}$ and $C_{j}$ denote the sensitivity and specificity of test *j*, respectively.

To estimate the model parameters we formulate the model within the Bayesian framework of inference and use Markov chain Monte Carlo (MCMC) simulation for model fit. The following prior distributions were adopted for the parameters:

$\mu_{k}^{d}\text{ }\sim\text{ }N\left(0,100\right)$, $\tau_{k}^{d}=\frac{1}{\sigma_{kk}^{d}}\text{ }\sim\text{ }Gamma(0.01,0.01)$, $\rho_{kk}^{d}\text{ }\sim\text{ }Uniform(-1,1)$ and

$\pi\text{ }\sim\text{ }Beta(\alpha,\beta)$. *α* and *β* can be re-written in terms of mean ($m_{\pi}$) and variance ($\sigma_{\pi}^{2}$) with $\alpha=m_{\pi}\left(\frac{m_{\pi}\left(1-m_{\pi}\right)}{\sigma_{\pi}^{2}}-1\right),\text{ }\beta=\left(1-m_{\pi}\right)\left(\frac{m_{\pi}\left(1-m_{\pi}\right)}{\sigma_{\pi}^{2}}-1\right)$

Based on our previous observations we have set $m_{\pi}$ to 0.1 and $\sigma_{\pi}^{2}$ to 0.05.

In addition, we assume $S_{j}\sim Beta\left(\alpha_{j}^{S},\beta_{j}^{S}\right),j=1,...,4$ and $C_{j}\sim Beta\left(\alpha_{j}^{C},\beta_{j}^{C}\right),j=1,2,3$ and $C_{4}=1$. Again, $\alpha_{j}^{S}$ and $\beta_{j}^{S}$ or $\alpha_{j}^{C}$ and $\beta_{j}^{C}$ can be re-written in terms of means ($m_{j}^{S}$,$m_{j}^{C}$) and variances [$\left(\sigma_{j}^{S}\right)^{2}$,$\left(\sigma_{j}^{C}\right)^{2}$]. We assigned means and variances as indicated in table I. Initial means for the individual test sensitivities and specificities were based on previously published estimates (see References 9–12, 34–38, 45, 46 in main text).

The models were fitted in WinBUGS and convergence was achieved before 30000 iterations. Convergence was assessed informally by inspection of the ergodic averages of selected parameters.
