# Supplementary material for: Bayesian Receiver Operating Characteristic Estimation of Multiple Tests for Diagnosis of Bovine Tuberculosis in Chadian Cattle
Source: PLoS One. 2009 Dec 9;4(12):e8215. doi: 10.1371/journal.pone.0008215 (PMC2785429; doi:10.1371/journal.pone.0008215)
Supplement: Text S2 — WinBUGS code (0.03 MB DOC) [file pone.0008215.s003.doc]

**Text S2 – WinBUGS code**

(For model 2A; Table S1)

model {

for (i in 1:929) {

tuber[i] ~ dnorm(mu1[i],tau1[i]) # test 1

sentry100[i] ~ dnorm(condmu[i],condtau[i]) # test 2 (test2 |test1)

genios[i]~ dnorm(condmu1[i],condtau1[i]) # test 3 (test3 |test2,test1)

T[i]~dbern(prev_true) # latent disease state

# mean and precision for the test values of test 1:

mu1[i] <- lambda1[TT[i]]

tau1[i] <- gamma1[TT[i]]

# conditional mean and precision for the test values of test 2:

condmu[i] <- lambda2[TT[i]]+rho12[TT[i]]*sqrt(gamma1[TT[i]]/gamma2[TT[i]])*(tuber[i]-lambda1[TT[i]])

condtau[i] <- (gamma2[TT[i]])/(1-pow(rho12[TT[i]],2))

# conditional mean and precision for the test values of test 3:

condmu1[i] <- lambda3[TT[i]]+(1./det[TT[i]])*((rho32[TT[i]]+rho12[TT[i]]*rho31[TT[i]])*(1./(gamma1[TT[i]]*sqrt(gamma2[TT[i]]*gamma2[TT[i]])))*(sentry100[i]-lambda2[TT[i]])+(rho31[TT[i]]-rho12[TT[i]]*rho32[TT[i]])*(1./(gamma2[TT[i]]*sqrt(gamma1[TT[i]]*gamma3[TT[i]])))*(tuber[i]-lambda1[TT[i]]))

condtau1[i] <- 1./((1/gamma3[TT[i]])-((pow(rho32[TT[i]],2)+pow(rho31[TT[i]],2))/(det[TT[i]]*gamma1[TT[i]]*gamma2[TT[i]]*gamma3[TT[i]])))

}

# considering the 100% specificity of PCR for the latent disease status

for (i in 1:929) {

TT[i]<-equals(truinf[i],0)*(2-T[i])+equals(truinf[i],1)

}

det[1]<-(1-pow(rho12[1],2))/(gamma1[1]*gamma2[1])

det[2]<-(1-pow(rho12[2],2))/(gamma1[2]*gamma2[2])

prev_true~dbeta(a,b) # prior for the true disease prevalence

a<-(m*m*(1-m)/s) -m

b<-(a/m)-a

# prior for the mean of test 1-3 for the diseased and non-diseased population:

lambda1[1] ~ dnorm(0,0.01)I(lambda1[2],)

lambda2[1] ~ dnorm(0,0.01)I(lambda2[2],)

lambda3[1] ~ dnorm(0,0.01)

lambda1[2] ~ dnorm(0,0.01)

lambda2[2] ~ dnorm(0,0.01)

lambda3[2] ~ dnorm(0,0.01)

# prior for the correlation coefficients:

rho12[1] ~ dunif(-1,1)

rho12[2] ~ dunif(-1,1)

rho31[1] ~ dunif(-1,1)

rho31[2] ~ dunif(-1,1)

rho32[1] ~ dunif(-1,1)

rho32[2] ~ dunif(-1,1)

# prior for the precision of test 1-3 for the diseased and non-diseased population:

gamma1[1] ~ dgamma(0.01,0.01)

gamma2[1] ~ dgamma(0.01,0.01)

gamma3[1] ~ dgamma(0.01,0.01)

gamma1[2] ~ dgamma(0.01,0.01)

gamma2[2] ~ dgamma(0.01,0.01)

gamma3[2] ~ dgamma(0.01,0.01)

# variance of test 1-3 for the diseased and non-diseased population:

sigma1[1] <- 1/gamma1[1]

sigma1[2] <- 1/gamma1[2]

sigma2[1] <- 1/gamma2[1]

sigma2[2] <- 1/gamma2[2]

sigma3[1] <- 1/gamma3[1]

sigma3[2] <- 1/gamma3[2]

# AUC for each test :

AUC1 <- phi(-(lambda1[2]-lambda1[1])/sqrt(sigma1[2]+sigma1[1]))

AUC2 <- phi(-(lambda2[2]-lambda2[1])/sqrt(sigma2[2]+sigma2[1]))

AUC3 <- phi(-(lambda3[2]-lambda3[1])/sqrt(sigma3[2]+sigma3[1]))

# Sensitivities/specificities for each test for given cut-off values

Se1 <- 1-phi((2.5-lambda1[1])/sqrt(sigma1[1]))

Sp1 <- phi((2.5-lambda1[2])/sqrt(sigma1[2]))

Se1OIE <- 1-phi((4.5-lambda1[1])/sqrt(sigma1[1]))

Sp1OIE <- phi((4.5-lambda1[2])/sqrt(sigma1[2]))

Se2 <- 1-phi((15-lambda2[1])/sqrt(sigma2[1]))

Sp2 <- phi((15-lambda2[2])/sqrt(sigma2[2]))

Se3 <- 1-phi((38-lambda3[1])/sqrt(sigma3[1]))

Sp3 <- phi((38-lambda3[2])/sqrt(sigma3[2]))

}
